# Ectonucleotidases in Acute and Chronic Inflammation

**DOI:** 10.3389/fphar.2020.619458

**Published:** 2021-02-03

**Authors:** Anna Lisa Giuliani, Alba Clara Sarti, Francesco Di Virgilio

**Affiliations:** Section of Experimental Medicine, Department of Medical Sciences, University of Ferrara, Ferrara, Italy

**Keywords:** ecto-nucleotidases, ATP, purinergic receptors, immune cells, acute inflammation, chronic inflammatory diseases, tumors

## Abstract

Ectonucleotidases are extracellular enzymes with a pivotal role in inflammation that hydrolyse extracellular purine and pyrimidine nucleotides, e.g., ATP, UTP, ADP, UDP, AMP and NAD^+^. Ectonucleotidases, expressed by virtually all cell types, immune cells included, either as plasma membrane-associated or secreted enzymes, are classified into four main families: 1) nucleoside triphosphate diphosphohydrolases (NTPDases), 2) nicotinamide adenine dinucleotide glycohydrolase (NAD glycohydrolase/ADP-ribosyl cyclase/cyclic ADP-ribose hydrolase 1), 3) ecto-5′-nucleotidase (NT5E), and 4) ecto-nucleotide pyrophosphatase/phosphodiesterases (NPPs). Concentration of ATP, UTP and NAD^+^ can be increased in the extracellular space thanks to un-regulated, e.g., cell damage or cell death, or regulated processes. Regulated processes include secretory exocytosis, connexin or pannexin hemichannels, ATP binding cassette (ABC) transporters, calcium homeostasis modulator (CALMH) channels, the ATP-gated P2X7 receptor, maxi-anion channels (MACs) and volume regulated ion channels (VRACs). Hydrolysis of extracellular purine nucleotides generates adenosine, an important immunosuppressant. Extracellular nucleotides and nucleosides initiate or dampen inflammation via P2 and P1 receptors, respectively. All these agents, depending on their level of expression or activation and on the agonist concentration, are potent modulators of inflammation and key promoters of host defences, immune cells activation, pathogen clearance, tissue repair and regeneration. Thus, their knowledge is of great importance for a full understanding of the pathophysiology of acute and chronic inflammatory diseases. A selection of these pathologies will be briefly discussed here.

## Introduction

It is thought that ATP might be the most ancient extracellular messenger used by primordial cells to send messages to their neighbours, or simply as a passive signal of danger or distress (Verkhratsky et al., 2020). Every messenger system requires the messenger (i.e. ATP), antennae that recognize and decode the messenger (i.e. purinergic receptors), and a mechanism to stop the signal and prevent over-stimulation or receptor desensitization (i.e. nucleotidases). Therefore, it is likely that ectonucleotidases appeared early in evolution as close cell-to-cell communication partners of ATP and the early purinergic receptors, likely of the P2X subtype (Verkhratsky et al., 2020). Now ectonucleotidases are found in virtually all mammalian tissues, and homologues have been even identified in platyhelmints (*Schistosoma mansoni*), where they exhibit a similar enzyme activity, i.e. ATP and possibly NAD^+^ hydrolysis, as the mammalian enzymes (Ferrero et al., 2019). Thus, ectonucleotidases are an indispensable enzyme system and an appealing target of innovative therapy.

## Ectonucleotidases Set Extracellular Nucleotide and Nucleoside Levels

**FIGURE 1:**
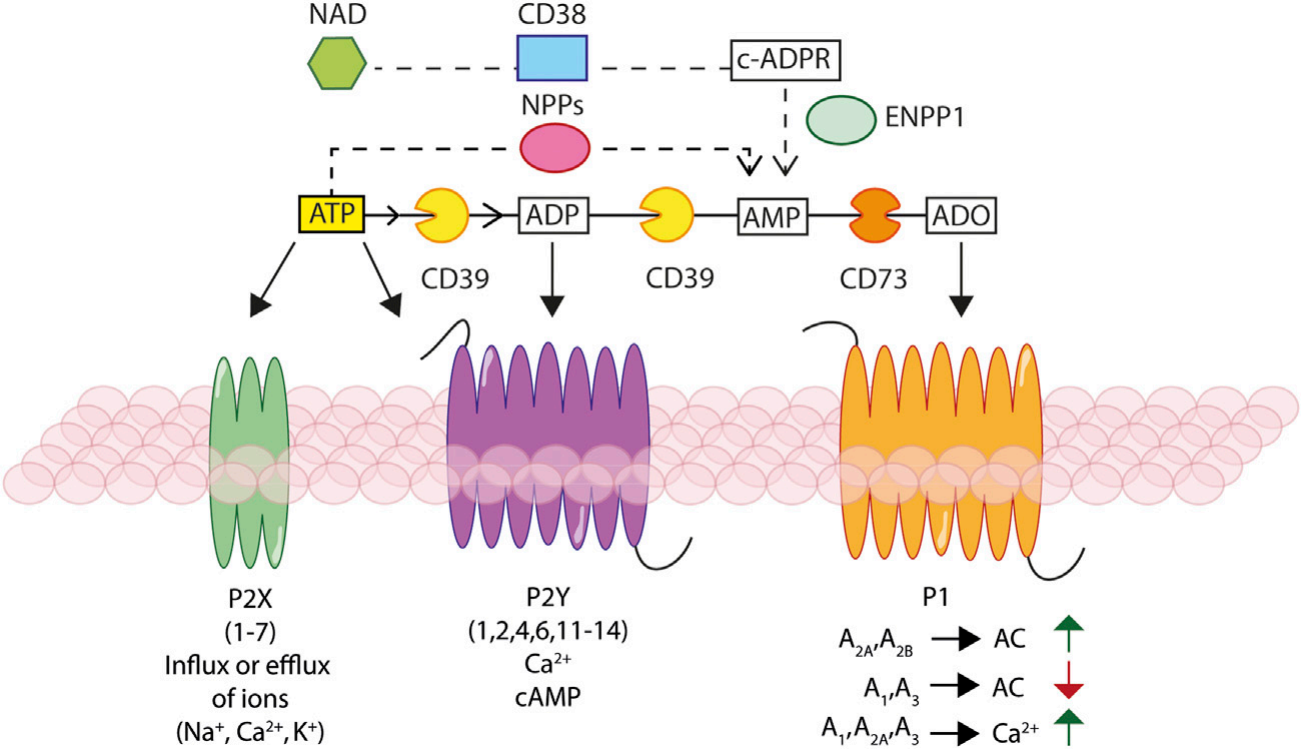
Schematic rendition of the basic elements of the purinergic signalling. Ectonucleotidases, NTPDase1/CD39, NAD glycohydrolase/CD38, NPPs and NT5E/CD73, hydrolyse extracellular ATP and NAD^+^, generating ADP, AMP, and adenosine (ADO). Extracellular ATP and ADP activate different P2X ionotropic and/or P2Y metabotropic receptors, leading to changes in the intracellular ion and/or cAMP concentration. Extracellular ADO stimulates P1 receptors responsible of modulation of adenylate cyclase (AC) activity and leading to changes in cAMP and Ca^2+^ concentration.

Ectonucleotidases are enzymes designated to hydrolyse extracellular nucleotides, mainly ATP, UTP, and NAD^+^, which generate metabolites relevant to immune and inflammatory responses. They are expressed at high level by cells of the immune system, mainly at the cell surface, and classified into four major families: ecto-nucleoside triphosphate diphosphohydrolases (NTPDases) (EC 3.6.1.5), nicotinamide adenine dinucleotide glycohydrolase (NAD glycohydrolase/ADP-rybosil cyclase/cyclic ADP-ribose hydrolase 1)‐ ecto-5′-nucleotidase (NT5E/CD73) (EC 3.1.3.5), and ecto-nucleotide pyrophosphatase/phosphodiesterases (NPPs) (Zimmermann et al., 2012; Linden et al., 2019) (Table 1) (Figure 1). In addition, nucleoside diphosphate kinase (NDPK), adenylate kinase (AK) and ecto-F1-F0 ATP synthase also participate in the control of extracellular nucleotide levels (Moser et al., 2001).

**TABLE 1:** Main ectonucleotidases and ecto-enzymes involved in regulation of purinergic signalling.

Ecto-enzyme	Substrates	Products	Expression	Function	Ref
NTPDase1/CD39	ATP, ADP	ADP, AMP	Monocytes, DCs, NK cells, Treg cells, ECs	Converts ATP/ADP to AMP	Mizumoto et al. (2002); Deaglio et al. (2007); Deaglio and Robson (2011); Kishore et al. (2018)
NAD glycohydrolase/CD38	NAD+, cADPR	cADPR, AMP	Activated T and B cells, plasma cells, DCs	Converts NAD^+^ to cADP ribose	Linden et al. (2019)
NPP1	ATP, ADP, NAD+	AMP	Inflammatory cells	Production of AMP starting from different substrates	Wang et al. (2018a)
NPP2	LPC, ATP	LPA, AMP	Inflammatory and tumor cells	Converts LPC to LPA	Sevastou et al. (2013)
NT5E/CD73	AMP	Adenosine	Follicular DCs, ECs, T and B lymphocytes	Converts AMP to adenosine	Thomson et al. (1990); Bono et al. (2015); Antonioli et al. (2016)
ADA	Adenosine	Inosine	DCs and lymphocytes	Converts adenosine to inosine	Franco et al. (1998); Desrosiers et al. (2007)

LPC, Lysophosphatidylcholine; LPA, lysophosphatidic acid; ECs, endothelial cells; DCs, dendritic cells; ADA, adenosine deaminase.

NTPDases, basically expressed in all tissues (Robson et al., 2006; Yegutkin, 2008; Kukulski et al., 2011b), hydrolyse nucleoside triphosphates and diphosphates producing nucleoside monophosphates (Table 1). Eight members of this family have been identified so far in mammals. Of these, NTPDase1/CD39, NTPDase2/CD39L1, NTPDase3/CD39L3, and NTPDase8/hATPDase are expressed on the cell surface, NTPDases 4–7 are present in intracellular organelles, while NTPDases 5 and 6 are also found as secreted forms (Robson et al., 2006; Knowles, 2011). The NTPDases are very likely the most important extracellular nucleotide-hydrolysing enzymes. Optimal activity requires millimolar concentrations of Ca^2+^ or Mg^2+^ and a pH in the 7–8 range. The NTPDases expressed on the plasma membrane, i.e. NTPDases 1, 2, 3, and 8, hydrolyse both nucleoside triphosphates and diphosphates, while the other members of the family show a more restricted substrate selectivity. Thus, extracellular ATP, UTP, ADP, and UDP are hydrolysed to AMP and UMP. NTPDase1/CD39 is the best characterized ectonucleotidase, widely expressed on different immune cell types, e.g., monocytes, dendritic cells (DCs), T regulatory (Treg) cells and natural killer (NK) cells, besides vascular endothelial cells (Mizumoto et al., 2002; Deaglio et al., 2007; Deaglio and Robson, 2011; Kishore et al., 2018). NTPDase activity has been found in blood circulating microparticles (MPs) (Jiang et al., 2014). MPs, produced and released by different cell types, act at intercellular level as vehicles for cell-to-cell transfer of enzymes, receptors and miRNAs. MP-associated NTPDase activity was found to dampen endothelial cell activation by modulating exchange of regulatory signals between leucocytes and vascular cells (Banz et al., 2008).

The NAD glycohydrolase/CD38, a cell surface glycoprotein expressed in thymocytes, activated peripheral blood T and B cells, plasma cells, and DCs, hydrolyses NAD^+^ to cyclic-ADP ribose (cADPR) (Table 1). Since it can be released in a soluble form or can be internalized, NAD glycohydrolase/CD38 has likely both an extracellular and an intracellular function. In fact, cADPR is an intracellular messenger triggering Ca^2+^ release from intracellular stores. Extracellular cADPR on the contrary is converted to AMP by the NAD glycohydrolase/CD38 itself, or by NPP1. AMP is eventually degraded to adenosine by NT5E/CD73. In mice, NAD glycohydrolase/CD38 is necessary for migration of mature DCs to secondary lymphoid tissues, and accordingly NAD glycohydrolase/CD38 deficiency results in impairment of soluble immunity to T cell–dependent antigens (Wykes et al., 2004). Dysregulation of NAD glycohydrolase/CD38 has been implicated in several inflammatory pathologies such as diabetes, heart disease, asthma and cancer (Linden et al., 2019). The combined NAD glycohydrolase/CD38-NT5E/CD73 activity is very important for the generation of immunosuppressive adenosine at inflammatory sites and in the tumor microenvironment (TME). Hydrolysis of extracellular NAD^+^ affects the immune response in multiple ways, including a protective activity on Treg and NK cells (Linden et al., 2019). By removing extracellular NAD^+^, NAD glycohydrolase/CD38 inhibits ADP-ribosyltransferase 2.2 (ART2.2), an ectoenzyme that transfers ADP-ribose from NAD^+^ to the P2X7 receptor (P2X7R), thus lowering the activation threshold of this receptor by extracellular ATP and facilitating apoptosis (Adriouch et al., 2008; Scheuplein et al., 2009; Schwarz et al., 2009). This mechanism, however, is only active in mice as human T lymphocytes lack ART2.2.

A key role in purinergic signalling is played by NT5E/CD73, the main enzyme producing extracellular adenosine from AMP. Although in several tissues phosphatases contribute to conversion of AMP to adenosine, NT5E/CD73 is the dominant adenosine-generating enzyme. NT5E/CD73 has been described both as a Zn^2+^-binding glycosylphosphatidylinositol (GPI)-anchored, extracellularly oriented, homo-dimeric protein, and as a soluble form (Airas et al., 1997; Yegutkin et al., 2000). The two 70-kD subunits host binding sites for catalytic ions at the N-terminal domain, and an AMP binding site at the C-terminal domain. NT5E/CD73 is expressed by stromal cells, follicular DCs, endothelial cells, neutrophils, macrophages and by subpopulations of human T lymphocytes (Bono et al., 2015) (Table 1). Soluble NT5E/CD73, mainly shed from endothelial cells and lymphocytes, is present both in serum and lymph in healthy conditions (Yegutkin et al., 2015), but its concentration increases during inflammation (Schneider et al., 2019). NT5E/CD73 hydrolyzes both ribo- and deoxyribo-nucleoside 5′-monophosphates, among which AMP with high affinity, and CMP, UMP, IMP, and GMP with low affinity. ADP binds to the catalytic site of NT5E/CD73 but is not hydrolysed, therefore acting as competitive inhibitor (Naito and Lowenstein, 1981). ADP generated from released ATP inhibits NT5E/CD73 and delays adenosine formation, ultimately promoting inflammation (Vieira et al., 2014). Hydrolysis of extracellular ADP by other ectonucleotidases is therefore needed to prevent NT5E/CD73 inhibition.

NPPs are ecto-enzymes that hydrolyse a wide range of substrates (Table 1). The NPP family includes seven members, NPP1–7, according to their order of cloning. NPP1–3 hydrolyse pyrophosphate or phosphodiester bonds in a wide variety of substrates, e.g., nucleoside triphosphates and diphosphates, NAD^+^, FAD, UDP-sugars, and di-nucleoside polyphosphates (Stefan et al., 2006). NPP2, also named autotaxin, hydrolyses phospholipids more efficiently than nucleotides, acting as a lysophospholipase D, to generate the bioactive phospholipid mediators lysophosphatidic acid (LPA) and sphingosine-1-phosphate (S1P) (Umezu-Goto et al., 2002). LPA and S1P promote a variety of cell responses, among which migration, proliferation, tumor cell survival and angiogenesis (Valdes-Rives and Gonzalez-Arenas, 2017). The NPP2–LPA axis has been implicated in various physiological and pathological pathways, including chronic inflammatory diseases such as multiple sclerosis, rheumatoid arthritis, hepatitis and pulmonary fibrosis (Sevastou et al., 2013). NPP6 and 7 hydrolytic activity is restricted to phospholipids, whereas catalytic properties of NPP4 and 5 remain unknown.

In addition to adenosine-producing ecto-enzymes, an important component of the extracellular purine-inactivating chain is adenosine deaminase (ADA) which catalyses the deamination of adenosine to inosine (Table 1). ADA is widely expressed in different tissues such as thymus, spleen, intestine and other non-lymphoid tissues (Moriwaki et al., 1999; Spychala, 2000), and is also present as an ecto-enzyme on the plasma membrane of DCs (Desrosiers et al., 2007) and lymphocytes (Franco et al., 1998).

Extracellular ATP is sequentially hydrolysed to ADP and AMP by NTPDase1/CD39, or can be directly hydrolysed to AMP by NPPs. AMP can be also generated from NAD^+^ via sequential activity of NAD glycohydrolase/CD38 and NPP1 (Linden et al., 2019), and is catabolized to adenosine by NT5E/CD73. In conclusion, ectonucleotidases regulate the extracellular concentration of ATP, NAD^+^ and other nucleotides, and their conversion into several bioactive metabolites (Giuliani et al., 2018; Boison and Yegutkin, 2019).

## The Different Pathways Responsible for Nucleotide Release

**FIGURE 2:**
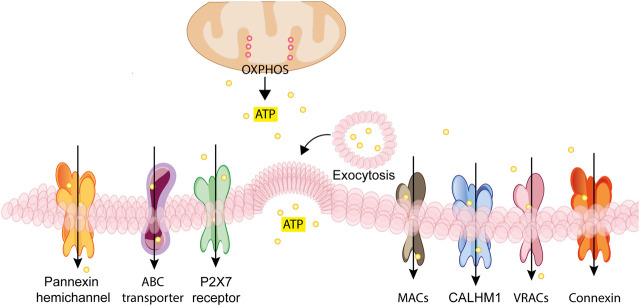
Schematic rendition of the different pathways for regulated nucleotide release. ATP generated inside the cell by glycolysis and oxidative phosphorylation (OXPHOS) can be released through vesicular exocytosis, connexin or pannexin channels, specific ATP binding cassette (ABC) transporters, calcium homeostasis modulators (CALHM) channels, the P2X7 receptor, maxi-anion channels (MACs) or through volume regulated ion channels (VRACs). These different pathways variably participate in ATP release in various cell types depending on the given patho-physiological context.

ATP, UTP, ADP, and NAD^+^ are released into the extracellular space via either un-regulated, e.g., cell damage or death, or regulated mechanisms (Lazarowski et al., 2011; Burnstock, 2012). The mechanisms responsible for controlled release include secretory exocytosis, connexin or pannexin hemichannels (Lohman and Isakson, 2014; Dahl, 2015), ATP binding cassette (ABC) transporters, calcium homeostasis modulator (CALMH) channels, the ATP-gated P2X7R (Pellegatti et al., 2005; Suadicani et al., 2006) and two classes of channels relevant for maintenance of normal intracellular osmolarity, i.e. maxi-anion channels (MACs) and volume regulated ion channels (VRACs) (Taruno, 2018) (Figure 2).

Regulated exocytosis is a main mechanism driving ATP release from intact cells (Imura et al., 2013). ATP storage inside exocytotic vesicles is due to a vesicular nucleotide transporter (VNUT) localized on the membrane of secretory vesicles (Sawada et al., 2008; Miyaji et al., 2011). VNUT accumulates ATP into the lumen exploiting the proton-dependent electrochemical gradient established by a vacuolar-ATPase (v-ATPase) (Nelson et al., 2000). Fusion of the exocytotic vesicles with the plasma membrane by the soluble N-ethylmaleimide-sensitive factor attachment protein receptor (SNARE)-mediated route (Sudhof and Rothman, 2009) ultimately allows the release of nucleotides into the extracellular space (Martens and McMahon, 2008; Moriyama et al., 2017).

Intercellular exchange of ions and small molecules occurs via gap-junction channels formed by innexins in invertebrates and connexins in vertebrates. Vertebrates also express innexin homologs, e.g., the pannexins, which make channels, usually hemi-channels. Although connexins and pannexins have no significant sequence homology, they share similarities in quaternary structure and in membrane topology (Beyer and Berthoud, 2018). The N- and C-terminal domains are localized on the cytoplasmic side of the plasma membrane, four stretches span the plasma membrane, and two loop domains are present on the cytoplasmic and the extracellular side (D'Hondt et al., 2009; Scemes et al., 2009). While pannexins only form hemichannels, connexins can assemble as both gap junctions and hemichannels (Sosinsky et al., 2011; Lohman and Isakson, 2014; Dahl, 2015). The hexameric membrane structures formed by assembly of connexins or pannexins, respectively named connexons and pannexons, allow small cation (e.g., Na^+^ and Ca^2+^) influx (Baroja-Mazo et al., 2013; Penuela et al., 2013), as well as transit of molecules of MW up to 1–2 kDa, such as ATP, glutamate and prostaglandins (Bao et al., 2004; Kang et al., 2008). Connexin-43 and pannexin-1 are thought to be the main gap junction-like channels involved in ATP release (Junger, 2011).

Connexins participate in intercellular communication in various physiological and pathological settings such as cell growth and differentiation, endocrine and exocrine secretion, immune response, inflammation and tumors (Mese et al., 2007; Herve and Derangeon, 2013; Leybaert et al., 2017; Villanelo et al., 2017; Wong et al., 2017; Cocozzelli and White, 2019). Connexins are classified according to the MW of their basic subunit, of which 21 isoforms are known in humans (Kar et al., 2012). Gap junctions established by connexons allow direct communication between the cytoplasm of adjacent cells, while undocked connexin hemichannels allow release of low MW cytoplasmic components into the extracellular milieu (Begandt et al., 2017; Belousov et al., 2017; Leybaert et al., 2017). Connexin hemichannels are very likely to be in the closed state in resting cells, transitioning to an open state in response to different stimulating agents (Wang et al., 2013a). Connexin-43, -37, -26 and -36 have been shown to support ATP release (Wang et al., 2013b), but a preeminent role is played by connexin-43 activated by increases in the intracellular Ca^2+^ concentration, plasma membrane depolarization, reactive oxygen species (ROS) or nitric oxide (NO). Connexin-43 can also be activated upon interaction of monocyte/macrophage Toll like receptor (TLR) 2 or TLR4 with the chemotactic factor N-formyl Met-Leu-Phe (fMLP) or lipopolysaccharide (LPS), respectively (Eltzschig et al., 2006; Wang et al., 2017).

The human pannexin family is comprised of three members, pannexin-1, -2 and -3 (Wang et al., 2013b). Pannexin-1 and -3 are widely expressed in different tissues while pannexin-2 is almost exclusively present in the brain (Penuela et al., 2013). In resting cells, pannexin channels are in a closed state, very likely due to the C-terminal tail that blocks the pore from the intracellular side (Dourado et al., 2014). In fact, C-terminal cleavage by caspase-3, -7 or -11 allows pannexin-1 channel opening (Sandilos et al., 2012; Yang et al., 2015). Thereafter, channel size progressively increases thanks to addition of further C-terminal tail-cleaved pannexin-1 subunits. Thanks to pannexons-induced increased permeability, molecules of size larger than ions, i.e. nucleotides, can cross the plasma membrane (Chiu et al., 2017). Various stimuli, such as intracellular calcium increase (Locovei et al., 2006), redox potential changes (Retamal, 2014), mechanical stress (Bao et al., 2004) and activation of the P2X7R (Iglesias et al., 2008; Pelegrin and Surprenant, 2009) can trigger pannexin-1 channel opening. An additional mode of pannexin-1 regulation is represented by internalization of the pannexin-1 channel itself, in an autocrine negative feedback loop driven by ATP-induced P2X7R activation (Boyce and Swayne, 2017). ATP and UTP released from apoptotic cells through pannexin-1 (Qu et al., 2011) promote monocyte recruitment (Elliott et al., 2009) and support NLRP3 inflammasome-driven IL-1β release in monocytes/macrophages (Ayna et al., 2012).

ABC transporters are integral membrane proteins that allow ATP-dependent movement across the plasma membrane of various molecules, among which cholesterol, lipids and both hydrophobic and hydrophilic drugs (Lohman et al., 2012). The multiple drug resistance (MDR1) gene product P-glycoprotein is the ABC transporter most consistently implicated in ATP release in the past (Abraham et al., 1993).

The calcium homeostasis modulators (CALHM) family includes six members two of which (CALHM1 and 3) have been recently identified as relevant for ATP release (Taruno, 2018). CALHM1, a plasma membrane voltage-gated ion channel showing structural and functional similarities with connexins and pannexins (Siebert et al., 2013), is expressed in many different tissues such as brain (Ma et al., 2012), taste buds (Taruno et al., 2013; Taruno et al., 2017), airway epithelia (Workman et al., 2017), and bladder (Sana-Ur-Rehman et al., 2017). In addition, CALMH1/CALMH3 hexameric fast voltage-gated ATP-release channels have been recently identified in type II taste bud cells (Ma et al., 2018).

ATP release can also occur through a receptor for extracellular ATP belonging to the P2XR family, i.e. the P2X7R (Pellegatti et al., 2005; Suadicani et al., 2006), especially when this receptor is over-stimulated and the associated large conductance pore (the macropore) fully opened, thus allowing transit of molecules up to 900 Da (Ohshima et al., 2010; Brandao-Burch et al., 2012; Karasawa et al., 2017). Although participation of accessory molecules to the formation of the P2X7R macropore has long been debated (Pelegrin and Surprenant, 2006; Locovei et al., 2007), it is now generally thought that the macropore is intrinsic to the P2X7R (Karasawa et al., 2017; Di Virgilio et al., 2018c). This hypothesis is further supported by the recent finding that a truncated P2X7R form lacking both amino and carboxyl termini, and therefore in principle with a low chance of interaction with other intracellular components, is able to generate the macropore (Karasawa et al., 2017).

Maxi-anion channels (MACs) are ubiquitous, ATP-permeable, large conductance anion-selective channels with pharmacological properties distinct from those of other anion channels (Sabirov et al., 2016). Their molecular identity has remained unknown until the recent finding that solute carrier organic anion transporter family member 2A1 (SLCO2A1) is the MAC core subunit (Sabirov et al., 2017). Very recently, gene silencing study showed that four annexin family members are involved in regulation of MACs activity (Islam et al., 2020). MACs, inactive in resting cells, undergo activation in presence of various stimuli such as high glucose (Best, 2002), ischemia and/or hypoxia (Dutta et al., 2004; Liu et al., 2008). Participation of MACs to ATP release is supported by the finding that hypotonic cell swelling-induced ATP release is significantly reduced by RNA interference of SLCO2A1 in mouse mammary epithelial C127 cells, and, on the contrary, potentiated when SLCO2A1 is heterologously expressed in human embryonic HEK293 fibroblasts which lack endogenous SLCO2A1 expression (Sabirov et al., 2017). However, despite this evidence, ATP currents through reconstituted MACs have yet to be demonstrated. MACs have been proposed as pathways for ATP release in some tissues such as macula densa (Bell et al., 2003), ischemic astrocytes (Liu et al., 2008) and ischemic-re-perfused heart (Dutta et al., 2004; Sabirov et al., 2017; Okada et al., 2019).

Volume regulated ion channels (VRACs) are ubiquitous channels (Nilius et al., 1994) important for maintenance of intracellular osmotic balance. VRACs are activated in response to hypotonic cell swelling to restore normal cellular volume by allowing efflux of organic and inorganic anionic osmolytes. VRAC subunits have been recently identified as leucine-rich-repeat-containing 8A protein (LRRC8A) and other LRRC8 members (B, C, D, E) that aggregate to form heteromers. Each individual VRAC may be formed by three or more different LRRC8 subunits (Gaitan-Penas et al., 2016; Lutter et al., 2017). LRRC8 subunit composition determines substrate selectivity (Planells-Cases et al., 2015; Schober et al., 2017), inactivation kinetics (Voss et al., 2014), and conductance (Syeda et al., 2016). LRRC8 subunits have four membrane-spanning domains with cytosolic amino- and carboxyl-termini (Voss et al., 2014) and high sequence homology with pannexin-1, suggesting that also LRRC8 subunits may form hetero-hexameric channels (Abascal and Zardoya, 2012; Konig and Stauber, 2019). It is likely that different LRRC8 combinations and variable stoichiometry form different VRACs channels. Finally, additional component(s), beside LRRC8 subunits, have been suggested to intervene in VRAC channels formation (Okada et al., 2017). Currently, while direct electrophysiological measurement of ATP currents through VRACs has not been reported, release of ATP has been demonstrated with the luciferine-luciferase assay in *Xenopus* oocytes injected with cDNAs of LRRC8 subunits and exposed to hypotonic stress (Gaitan-Penas et al., 2016).

## Receptors for Extracellular Nucleotides and Nucleosides

Receptors for extracellular nucleotides and for adenosine are P2 receptors (P2Rs) and P1 receptors (P1Rs), respectively (Burnstock and Knight, 2004) (Figure 1). Seven ionotropic (P2XR1-7) and eight metabotropic (P2YR_1,2,4,6,11–14_) receptors for nucleotides and four adenosine receptors (A_1_, A_2A_, A_2B_, A_3_) have been identified and cloned in humans.

The P2XRs that are gated exclusively by ATP, form channels allowing Na^+^ and Ca^2+^ influx, and K^+^ efflux (North, 2002; 2016). At least three P2X subunits assemble to form hetero- (e.g., P2X2/3 and P2X1/5) or homo-trimeric (P2X7) channels (North, 2002). Each P2X subunit is characterised by two membrane-spanning domains (TM1 and TM2), a large ecto-domain and intracellular N- and C-termini (Di Virgilio et al., 2017). To trigger channel opening all the three ATP-binding sites present in the P2XR trimer need to be occupied (Bean, 1990). Among P2XRs, the P2X7R has a special place in inflammation since its stimulation promotes NLRP3 inflammasome and the associated IL-1β maturation and secretion (Giuliani et al., 2017; Adinolfi et al., 2018). The majority of P2X7R-dependent pro-inflammatory responses, among which extracellular ATP release, are due to the opening of the plasma membrane pore (macropore) that allows the non-selective passage of aqueous molecules of MW up to 900 Da. The macropore is now thought to be intrinsic to the P2X7R (Karasawa et al., 2017; Di Virgilio et al., 2018c), and potentially gated also by ligands other than ATP (Di Virgilio et al., 2018a). NAD^+^ is the best characterized non-ATP P2X7R agonist in mouse T lymphocytes. In these cells, NAD^+^ serves as an ADP-ribose donor to ADP-ribosylate the P2X7R at arginine 125, close to the ATP-binding pocket (Seman et al., 2003). This reaction, catalysed by the plasma membrane enzyme ART2.2 causes long-lasting activation of mouse P2X7R. Since increased NAD^+^ content characterizes inflammatory sites (Adriouch et al., 2007), it is suggested that NAD^+^ has a role in the pathophysiological mechanism of P2X7R activation. Very recently, P2X7R was also found in circulation in a shed form (sP2X7R) associated to MPs (Giuliani et al., 2019). Although sP2X7R function has not been assessed yet, a link to inflammation is witnessed by its correlation with serum levels of the acute phase reactant C-reactive protein (CRP) (Giuliani et al., 2019).

The P2YRs are G protein-coupled metabotropic receptors triggering downstream effector signalling pathways leading to changes in the intracellular Ca^2+^ or cyclic adenosine monophosphate (cAMP) concentration, or both (von Kugelgen and Harden, 2011). Eight P2YRs have been identified and characterized so far in mammals: P2YR_1-2_, P2YR_4_, P2YR_6_, P2YR_11–14_. Preferred agonists are ATP (P2YR_11_), ADP (P2YR_1_, P2YR_12_ and P2YR_13_), UTP (P2YR_2_ and P2YR_4_), UDP (P2YR_6_), UDP-glucose and UDP-galactose (P2YR_14_). P2YR_1_, P2YR_2_, P2YR_4_, and P2YR_6_ activate Gq and phospholipase C-β (PLC-β), thus leading to inositol 1,4,5-trisphosphate (IP3) and diacylglycerol (DAG) generation from phosphatidylinositol 4,5-bisphosphate (PI[4,5]P2). IP3 triggers Ca^2+^ release from intracellular stores, therefore increasing its cytoplasmic concentration, while DAG activates protein kinase C (PKC) (Zimmermann, 2016). G_i/o_ protein activation by P2YR_12–14_ inhibits adenylyl cyclase (AC), thus reducing intracellular cAMP levels. P2YR_11_ stimulation induces increase of intracellular Ca^2+^ and cAMP via activation of both Gq and Gs. Other recently identified P2YRs-engaged intracellular signalling pathways include activation of phosphatidylinositol- 4,5-bisphosphate 3-kinase γ (PI3K-γ), phospholipase C-β2 and -β3, inward rectifying K^+^ (GIRK) channels, G protein-coupled receptor (GPCR) kinases 2 and 3, Rho, and mitogen activated protein kinases (MAPKs) (von Kugelgen and Harden, 2011; Erb and Weisman, 2012).

Affinity of P2YRs for their ligands is variable, from high nanomolar to low micromolar, while P2XR affinity ranges from the low micromolar to the near millimolar level. Therefore, purinergic signalling is endowed with the ability to finely tune a multiplicity of cell functions depending on the cell type, the receptor subtype expressed and extracellular agonists concentration.

The P1R family includes four adenosine receptors (A_1_, A_2A_, A_2B_, and A_3_) (Carpenter and Lebon, 2017; Antonioli et al., 2018; Camici et al., 2018) coupled to changes in cAMP and Ca^2+^ levels (Wang et al., 2004; Borea et al., 2018). A_1_ and A_3_ receptors are coupled to G proteins of the G_i_, G_q_, and G_0_ family and drive Ca^2+^ release from intracellular stores. A_2A_ and A_2B_ receptors are coupled to G_s_ or G_q_ resulting in AC or PLC activation, respectively. In addition, all P1Rs stimulate the MAPK pathway, i.e. extracellular signal regulated kinase 1 (ERK1), ERK2, Jun N-terminal kinase (JNK), and p38-MAPK. Extracellular adenosine can also be internalised by all cells through two types of transporters, the equilibrative nucleoside transporters (ENTs) and the concentrative nucleoside transporters (CNTs) (Young, 2016; Pastor-Anglada and Perez-Torras, 2018) to stimulate various intracellular pathways, AMP-activated protein kinase, adenosine kinase and S-adenosyl homocysteine hydrolase included (Antonioli et al., 2013). Although it may depend on the concentration and the given P1R subtype engaged, on the whole adenosine mainly activates anti-inflammatory and immune suppressive responses, with prevalence of those addressed to restore tissue homeostasis (Antonioli et al., 2013). The immunosuppressant activity of adenosine relies on the inhibition of virtually all immune cell populations, such as T and B lymphocytes, NK cells, DCs, granulocytes, monocytes, and macrophages (Le Vraux et al., 1993; Nemeth et al., 2003; Ben Addi et al., 2008; Barletta et al., 2012).

In conclusion, extracellular ATP, UTP, NAD and their hydrolysis products, primarily ADP and UDP, play a well-established role as pro-inflammatory mediators acting at P2YRs and P2XRs, while the terminal, NT5E/CD73-generated, product adenosine acts at P1Rs to mainly suppress immunity.

## Expression and Activity of Ectonucleotidases on Immune Cells

Extracellular ATP is a ubiquitous damage-associated molecular pattern (DAMP), and thus a key inflammatory mediator (Pandolfi et al., 2016; Di Virgilio et al., 2018c; Denning et al., 2019). The extracellular ATP concentration at inflammatory sites is in the hundred micromolar range of concentration, vs. the low nanomolar levels found in healthy tissues (Pellegatti et al., 2008; Wilhelm et al., 2010; Barbera-Cremades et al., 2012). Ectonucleotidases play a fundamental role in setting the concentration of extracellular ATP and NAD^+^, and of their metabolites, thus tightly controlling the biochemical composition of the inflammatory environment. Therefore, it is not surprising that ectonucleotidases are expressed virtually by all immune cells in a cell- and tissue-dependent fashion (Resta et al., 1998). In addition, their expression can be modulated following exposure to stress, hypoxia or inflammatory cytokines (Ryzhov et al., 2014; Longhi et al., 2017).

**FIGURE 3:**
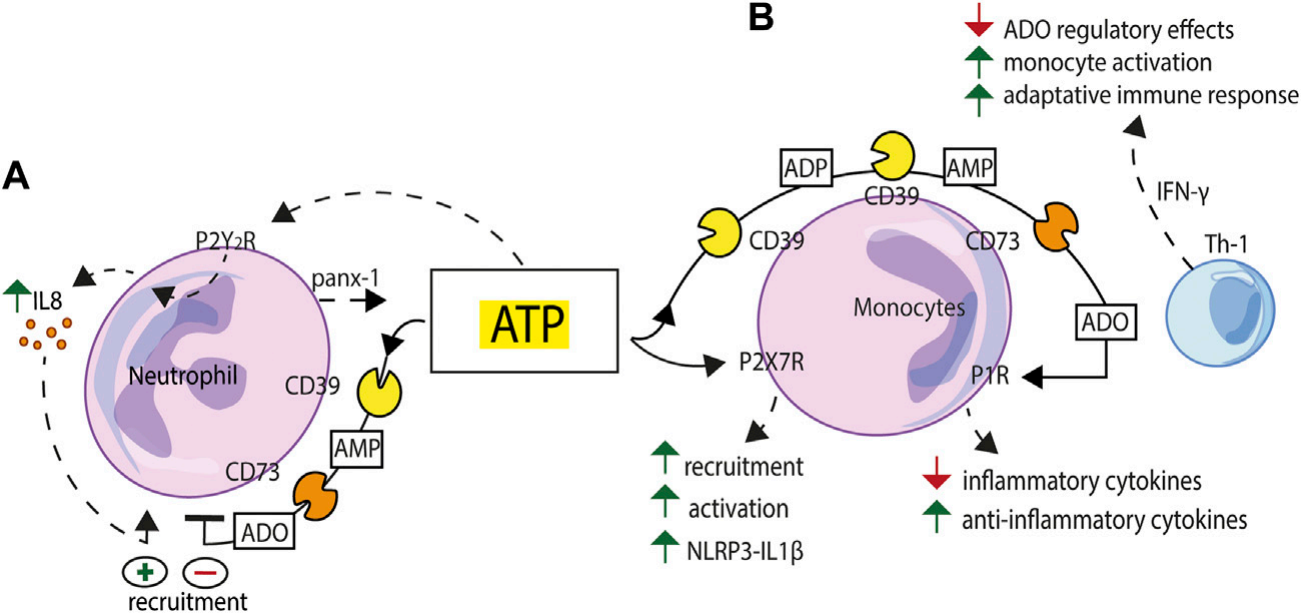
Schematic exemplification of purinergic receptor/ectonucleotidase cooperation in the activation/inhibition of the innate immune response. **(A)** ATP released via pannexin-1 (panx-1) from human neutrophils exposed to inflammatory stimuli triggers IL-8 production acting at the P2Y2R. NTPDase1/CD39 and NT5E/CD73 sequentially degrade extracellular ATP and limit neutrophil recruitment. **(B)** Extracellular nucleotides, firstly ATP, acting at the P2X7R, promote monocyte recruitment, modulate phagocytosis and support NLRP3 inflammasome-mediated IL-1β release. NTPDase1/CD39 and NT5E/CD73, expressed to high level on the macrophage plasma membrane, generate adenosine (ADO) and support a feed-back regulatory mechanism. Adenosine-mediated P1R stimulation generates an anti-inflammatory environment characterized by down-modulation of inflammatory cytokines release and enhanced secretion of anti-inflammatory cytokines and growth factors. This balance can be tilted towards an activated state, e.g., to support a more vigorous adaptive immune response, by IFN-γ, a stimulus that makes macrophages less sensitive to adenosine inhibition.

Neutrophils release ATP via pannexin-1 in response to inflammatory stimuli (Chen et al., 2015). Extracellular ATP in turn triggers IL-8 production from human neutrophils and neutrophil-like HL60 cells (Figure 3A). In LPS-stimulated human neutrophils, IL-8 release is markedly increased following NTPDase1/CD39 inhibition (Kukulski et al., 2011a). P2Y_2_R knockdown in HL60 cells decreases LPS-induced IL-8 production, suggesting a role for this receptor in neutrophil-driven inflammation. A role for NTPDase1/CD39 and NT5E/CD73 in attenuating *in vivo* neutrophil trafficking into the lungs during LPS-induced lung injury has been previously described (Reutershan et al., 2009).

A number of monocyte/macrophage functions are regulated by extracellular nucleotides and nucleosides. Extracellular ATP and UTP released from apoptotic cells mediate monocyte recruitment (Elliott et al., 2009), modulate phagocytosis (Soni et al., 2019; Zumerle et al., 2019), and support NLRP3 inflammasome-mediated IL-1β release (Ayna et al., 2012) (Figure 3B). Both NTPDase1/CD39 and NT5E/CD73 are expressed to high level in macrophages where they play a key role in the control of P2X7R-dependent responses and in the generation of adenosine (Levesque et al., 2010). Macrophage P1R stimulation by adenosine induces a regulatory state characterized by reduced release of inflammatory cytokines and enhanced secretion of anti-inflammatory cytokines and growth factors. The main function of this homeostatic system is to keep a transient macrophage activation state and prevent possible adverse effects due to prolonged macrophage activation. This balance can be tilted towards an activated state, for example to initiate and support a more vigorous adaptive immune response, by treating macrophages with IFN-γ, a stimulus that makes these cells less sensitive to the adenosine regulatory effects (Hamidzadeh and Mosser, 2016) (Figure 3B). Other cells present at inflammatory sites that express high ectonucleotidase levels, e.g., NTPDase1/CD39, such as mesenchymal stem cells (MSCs), also contribute to adenosine-based immunosuppressive mechanisms (de Oliveira Bravo et al., 2016). MSCs participate to the generation of an immunosuppressive microenvironment also by releasing NTPDase1/CD39-expressing extracellular vesicles (EVs). Macrophage phagocytosis is inhibited by MSC-derived EVs, an effect reverted by EVs pre-incubation with ectonucleotidases inhibitors (Katsuda et al., 2013). In addition, a soluble form of NT5E/CD73, which can be released from the plasma membrane by cleavage of its GPI anchor, can exert a remote control on the inflammatory microenvironment (Vieira et al., 2014).

**FIGURE 4:**
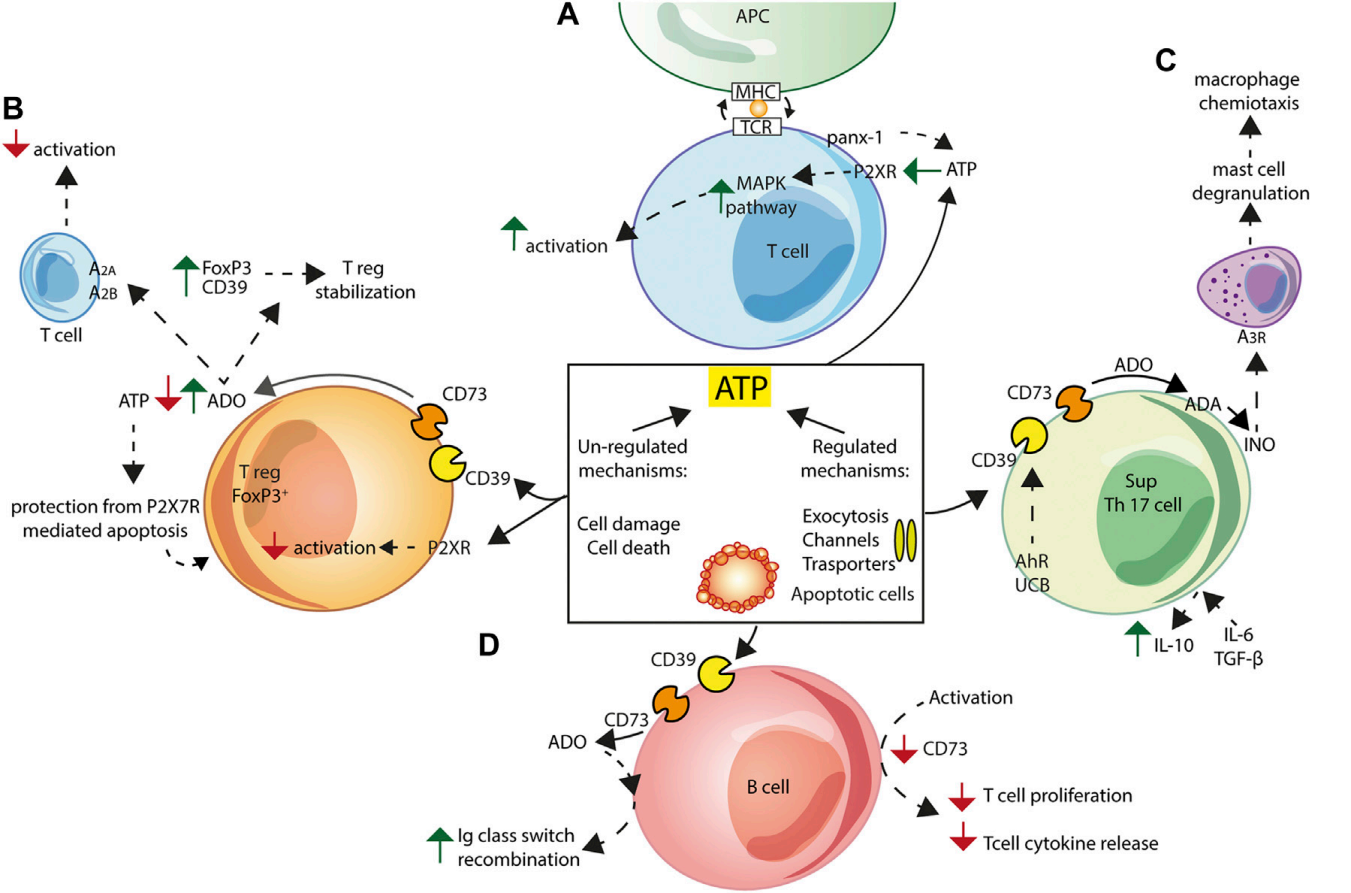
Schematic exemplification of purinergic receptor/ectonucleotidase cooperation in the activation/inhibition of the adaptive immune response. ATP can be released into the extracellular space via both regulated and non-regulated mechanisms. **(A)** In T cells, pannexin-1 (panx-1) and P2XRs localize at the immune synapse following T-cell receptor (TCR) engagement. ATP released via panx-1 triggers P2XRs activation leading to increased MAPK signalling and T cell activation. **(B)** Treg Foxp3^+^ cells generation and function are inhibited by ATP-mediated P2XRs signalling. Adenosine (ADO) formed by Treg NTPDase1/CD39 and NT5E/CD73 activity causes Treg cells stabilization, by increasing expression of Foxp3 and NTPDase1/CD39, and inhibits T cells responses acting at A_2A_ and A_2B_ adenosine receptors. Finally, Tregs are protected from P2X7R-mediated apoptosis thanks to extracellular ATP scavenging by NTPDase1/CD39 and NT5E/CD73. **(C)** Non-pathogenic Th17 cells express NTPDase1/CD39 and NT5E/CD73, and, following IL-6 and TGF-β stimulation, secrete IL-10, thus showing the typical Th17 suppressor cell (SupTh17) phenotype. NTPDase1/CD39 expression by Th17 lymphocytes is enhanced following exposure to aryl hydrocarbon receptor (AhR) agonists, such as unconjugated bilirubin (UCB). Enhanced SupTh17 adenosine deaminase (ADA) activity accelerates conversion of adenosine to inosine (INO), which activates the A_3_ adenosine receptor on mast cells, thus causing degranulation and release of macrophage chemotactic factors. **(D)** Human peripheral B cells co-express NTPDase1/CD39 and NT5E/CD73. *In vitro* activation of B lymphocytes co-cultured with T lymphocytes down-regulates NT5E/CD73 expression and inhibits T cell proliferation and T cell-dependent cytokine release. Extracellular adenosine contributes to immunoglobulin (Ig) class switch recombination in human naïve and IgM memory B cells.

T cells are important and active players in purinergic signalling. T-cell receptor (TCR) engagement promotes localization of pannexin-1 channels and P2XRs at the immune synapse. Autocrine ATP release triggers P2XRs activation, increases MAPK signalling and drives T cell activation (Schenk et al., 2008) (Figure 4A). On the contrary, P2XR-mediated signalling inhibits Treg cells generation and function (Schenk et al., 2011) (Figure 4B). Ectonucleotidases by setting extracellular ATP levels play a central role in the modulation of T cell responses. NTPDase1/CD39 and NT5E/CD73, both expressed on the surface of human FoxP3^+^ Tregs (9, 157), catalyse the generation of large amounts of adenosine that acts at A_2A_ and A_2B_ receptors to inhibit T cells responses (158). In addition, adenosine increases expression of both Foxp3 and NTPDase1/CD39, leading to Treg cells stabilization (Bao et al., 2016), and to the activation of an adenosine-producing positive feed-back loop (Ohta et al., 2012). Low expression of NT5E/CD73 on FoxP3^+^ Tregs might contribute to a dysregulated immune response in autoimmune diseases (Oliveira et al., 2015). Lastly, NTPDase1/CD39 and NT5E/CD73, by extracellular ATP scavenging, protect Treg cells from P2X7R-mediated apoptosis (Figure 4B).

Th17 cell responses are also tightly regulated by levels of extracellular nucleotides and nucleosides, and therefore by NTPDase1/CD39 and NT5E/CD73 activity (Doherty et al., 2012; Longhi et al., 2014) (Figure 4C). Th17 cells are classified into subpopulations that differ in their pathogenicity and ability to release cytokines and growth factors. Pathogenic Th17 cells secrete GM-CSF (El-Behi et al., 2011; Lee et al., 2012), while IL-6- and TGF-β-stimulated, non-pathogenic, Th17 cells secrete IL-10 and express NTPDase1/CD39 and NT5E/CD73 (Chalmin et al., 2012), therefore showing the typical Th17 suppressor (SupTh17) phenotype (Fernandez et al., 2016). NTPDase1/CD39 expression is enhanced following exposure to aryl hydrocarbon receptor (AhR) agonists, such as unconjugated bilirubin (UCB), with a known immune activity (Longhi et al., 2017). On the other hand, SupTh17 cells are resistant to the effects of adenosine as result of low expression of the A_2A_ adenosine receptor and accelerated adenosine catalysis by ADA (Longhi et al., 2014). High ADA activity of SupTh17 cells accelerates hydrolysis of adenosine to inosine, a pro-inflammatory nucleoside able to cause, via A_3_ adenosine receptor activation, mast cell degranulation (Jin et al., 1997) and the associated macrophage chemotaxis (Joos et al., 2017) (Figure 4C).

Human peripheral B cells co-express NTPDase1/CD39 and NT5E/CD73 and hydrolyse extracellular ATP to AMP and adenosine (Saze et al., 2013). Resting B cells in co-culture with T cells upregulate CD4^+^ and CD8^+^ T cells functions, while *in vitro*-activated B cells down-regulate NT5E/CD73 expression and inhibit T cell proliferation and T cell-dependent cytokine release, thus preventing the potentially harmful effects of activated T cells (Saze et al., 2013). In addition, extracellular adenosine critically contributes to immunoglobulin class switch recombination in human naive and IgM memory B cells, an essential process for mounting a protective humoral immune response (Schena et al., 2013) (Figure 4D).

## Anti-bacterial Acute Inflammatory Responses

Extracellular ATP is a DAMP released during sterile and septic inflammation to recruit specialized cells at inflammatory sites, thus ectonucleotidases have an important function to allow efficient pathogen clearance at septic foci. Extracellular adenosine produced by NT5E/CD73 suppresses macrophage antibacterial responses, thus impairing innate immune response against infectious agents (Costales et al., 2018). On the contrary, low NT5E/CD73 activity supports macrophage phagocytosis and an efficient clearance of internalized bacteria. NT5E/CD73 down-regulation or inhibition during *Salmonella* infection enhances production of pro-inflammatory cytokines and NO from macrophages and improves intracellular killing (Costales et al., 2018).

Neutrophil recruitment and activation are crucial for host defense in lung infection sustained by *Streptococcus pneumoniae.* However, in the late phases of the infection, neutrophil antimicrobial activity declines. This progressive exhaustion correlates with reduced NT5E/CD73 expression (Siwapornchai et al., 2020). Extracellular adenosine has an important role in *S. pneumoniae* killing as its production dramatically increases resistance to *S. pneumoniae* lung infection in mice; accordingly, NT5E/CD73-inhibition inhibits *in vitro* and *in vivo S. pneumoniae* killing by neutrophils (Bou Ghanem et al., 2015). Enhanced susceptibility of CD73^*−/−*^ mice to *S. pneumoniae* is reversed by neutrophil depletion, pointing to this cell type as the target of adenosine activity. It is apparently paradoxical that reduced NT5E/CD73 activity, which lowers extracellular adenosine levels, causes inhibition of neutrophil functions. This seems to be due to up-regulation of IL-10 release in the absence of NT5E/CD73 (Siwapornchai et al., 2020). In fact, pneumococcal infection up-regulates IL-10 production in CD73^*−/−*^ but not in WT mice (Siwapornchai et al., 2020).

Transgenic mice overexpressing human NTPDase1/CD39, under the control of the airway-specific Clara cell 10-kDa protein gene promoter, do not develop spontaneous lung inflammation, and following intra-tracheal instillation of LPS undergo accelerated recruitment of neutrophils and CD8^+^ T lymphocytes and B lymphocytes to the airways and delayed macrophage clearance. These transgenic mice show increased lung recruitment of neutrophils and macrophages upon *Pseudomonas aeruginosa* infection, and clear the bacterial infection with high efficiency (Theatre et al., 2012). Therefore, constant elevated NTPDase1/CD39 activity in lung epithelia does not cause inflammation but improves host response to acute LPS or *P. aeruginosa* exposure (Theatre et al., 2012).

NTPDase1/CD39 and NT5E/CD73 may also affect antibacterial response by modulating Treg activity (Vieyra-Lobato et al., 2018; Alam et al., 2020), while NTPDase1/CD39 is upregulated on both CD4^+^ and CD8^+^ Teff cells at sites of acute inflammation thus attenuating responses to bacterial infections (Raczkowski et al., 2018). NTPDase1/CD39, due to its ATP-scavenging activity, strongly modulates P2X7R-mediated pro-inflammatory responses. Therefore, while NTPDase1/CD39 expression limits P2X7R-mediated inflammation and attenuates sepsis-induced liver injury, NTPDase1/CD39 genetic deletion exacerbates sepsis-induced liver injury (Savio et al., 2017). Combination of a P2X7R antagonist and A_2A_ adenosine receptor agonist is hepato-protective in abdominal sepsis (Savio et al., 2017). P2X7R deletion or pharmacological P2X7R blockade, or extracellular ATP scavenging, in LPS-primed macrophages attenuated inflammation, largely preventing increased cytokine secretion and tissue damage (Li et al., 2017; Savio et al., 2017).

Overall, experiments with CD73^*−/−*^ mice, in which poly-microbial sepsis was induced following cecal ligation and puncture, support the view that adenosine is protective in sepsis (Hasko et al., 2011).

Sepsis is also characterized by increased platelet activation and formation of platelet-neutrophil aggregates that become trapped in the microvasculature. These events are not currently manageable by effective therapeutic strategies, therefore it has been proposed that targeting platelet NTPDase1/CD39 might prevent micro-thrombi formation. To this aim, a recombinant fusion protein (targ-CD39) was made consisting of a single-chain antibody against activated glycoprotein IIb/IIIa and the extracellular domain of NTPDase1/CD39 (Granja et al., 2019). Targ-CD39 efficiently decreased platelet-leukocyte-endothelium interaction, pro-inflammatory cytokines transcription, microvascular platelet-neutrophil aggregate sequestration, expression of activation markers on platelets and neutrophils, leukocyte extravasation, and organ damage (Granja et al., 2019). Targ-CD39 caused a stronger improvement of survival in an experimental model of sepsis compared to the NTPDase1/CD39 extracellular domain fused to a non-functional antibody (nontarg-CD39) (Granja et al., 2019).

## Chronic Inflammatory and Autoimmune Diseases

Chronic inflammatory diseases are characterized by varieties of immune system dysfunctions, many of them resulting in auto-aggressive responses. Among them, rheumatoid arthritis, systemic lupus erythematosus, inflammatory bowel diseases and type 2 diabetes are very common and burdened by high morbidity and mortality. The pathogenesis of these diseases mainly depends on dysfunctional responses of monocyte/macrophages, Treg, Th17 and B lymphocytes. Purinergic signalling and ectonucleotidase activity might also be implicated.

### Rheumatoid Arthritis

Rheumatoid arthritis (RA) is an autoimmune chronic disease characterized by inflammation and damage to different organs and tissues, particularly the peripheral joints. Joint inflammation and synovial hyperplasia, eventually progressing to cartilage and bone damage with deformity and disability, are a feature of RA. Purinergic signalling has been implicated in several joint diseases, RA included (Corciulo and Cronstein, 2019), but targeting different components of the purinergic system has provided variable results. It is known that overall adenosine accumulation is protective, although in some pathological conditions excess adenosine may cause tissue injury due to activation of low affinity A_2B_ adenosine receptors (Pinto-Cardoso et al., 2020). Therefore, enhanced ectonucleotidase and reduced ADA activity are in principle beneficial. Direct targeting of adenosine A_2A_ receptors is a current appealing therapeutic option for the treatment of rheumatic diseases (Cronstein and Sitkovsky, 2017). NT5E/CD73-deficient mice are significantly more susceptible to type II collagen (CII)-induced arthritis than WT mice, show increased accumulation of pro-inflammatory cytokines in the joints, increased Th1 cell responses, and marked joint damage (Chrobak et al., 2015). Peripheral blood lymphocytes from RA patients express increased NTPDase and decreased ADA activity, a finding that might be interpreted as a compensatory mechanism to preserve a safe level of immunosuppressive adenosine (Dos Santos Jaques et al., 2013). Accordingly, peripheral blood mononuclear cells from RA patients show enhanced A_2A_ or A_3_ adenosine receptors expression that inversely correlated with disease activity score. A_2A_ and A_3_ agonists inhibit matrix metalloproteinase-1 (MMP-1) and MMP-3 release (Varani et al., 2011; Ravani et al., 2017).

Foxp3^+^CD39^+^CD25^+^ T-cells showing high NTPDase1/CD39 and low NT5E/CD73 levels are recruited to the joints of RA patients, but they seem to be unable to dampen inflammation. These cells suppress IFN-γ and TNF-α production, but fail to control IL-17A secretion by Teff cells (Herrath et al., 2014).

A deregulated macrophage-T cell interaction is suggested to play a role in RA pathogenesis. T cell activity is differently affected by macrophages stimulated with either macrophage colony-stimulating factor (M-CSF/CSF-1) or granulocyte-macrophage colony-stimulating factor (GM-CSF/CSF-2) (Ohradanova-Repic et al., 2018). GM-CSF-stimulated macrophages show a typical M1 profile with elevated pro-inflammatory activity, while M-CSF-stimulated macrophages show an M2 immunosuppressive phenotype largely due to the expression of ectonucleotidases. In addition, various local stimuli further contribute to shaping macrophage phenotype. Pro-inflammatory Th1 cytokines, such as IFN-γ, or TLR ligands skew macrophages to the M1 phenotype with enhanced microbicidal and tumoricidal activity, while the Th2 cytokines IL-4 and IL-13 drive M2 macrophage differentiation. Stimulation with IL-10, transforming growth factor-β (TGF-β) or glucocorticoids generates highly immunosuppressive “M2-like” macrophages (Mantovani et al., 2004; Biswas and Mantovani, 2010; Murray and Wynn, 2011; Ohradanova-Repic et al., 2018). An unbalance towards the M1 phenotype is found in human and murine arthritic joints (Ohradanova-Repic et al., 2018). It has been suggested that targeted delivery of methotrexate (MTX) to the immunosuppressive NTPDase1/CD39^+^- and NT5E/CD73^+^-high macrophages might give better results in treating RA than the administration of MTX as such (Ohradanova-Repic et al., 2018). MTX is one of the most effective treatments for RA thanks to its ability to inhibit several enzymes involved in nucleotide metabolism, and to promote release into the extracellular space of both adenosine and ATP, which is then converted to adenosine (Cronstein and Sitkovsky, 2017; Cronstein and Aune, 2020). MTX non-responder patients expressed lower NTPDase1/CD39 levels than responders (Peres et al., 2018) and low NTPDase1/CD39 expression on Treg cells was proposed as a biomarker for resistance to MTX therapy in RA (Peres et al., 2015).

### Systemic Lupus Erythematosus

Systemic lupus erythematosus (SLE) is a systemic autoimmune disease characterized by multiple tissue and organ damage and inflammation as result of impaired immune tolerance, auto-antibody production, immune complex (IC) formation and deposition. Although SLE pathogenesis remains obscure, it is well known that both innate and adaptive immunity play a major role. Macrophages from SLE patients are defective in their ability to clear apoptotic cell debris, thus prolonging exposure of potential autoantigens to immune cells (Byrne et al., 2012). In addition, macrophage-mediated IC clearance, TLR-mediated nucleic acid recognition, and IFN-dependent signalling are defective (Byrne et al., 2012). Among auto-reactive antibodies produced in SLE, a relevant role is played by anti-double strand (dsDNA) antibodies, which bind monocyte/macrophage TLR4, and activate the NLRP3 inflammasome, with production of ROS (Zhang et al., 2016). T cells also play an important function by amplifying the immune response and by contributing to organ damage (Comte et al., 2015; Tsokos et al., 2016). Dysregulated B cell responses have been reported (Wang et al., 2018b), in particular with the expansion of B cell subsets showing up-modulation of chemokine receptors, consistent with migration to target tissues and correlated with defined clinical manifestations (Wang et al., 2019).

Increased circulating ATP levels have been measured in SLE patients (Becker et al., 2019b). Lymphocytes from SLE patients show increased NTPDase expression and activity and enhanced ADA activity, while on the contrary NT5E/CD73 expression is unchanged (Becker et al., 2019a). Increased NTPDase1/CD39 expression might be a compensatory mechanism to down-modulate inflammation in the presence of high ATP blood concentrations as those detected in SLE patients (Becker et al., 2019a). On the other side, elevated ADA activity might contribute to SLE pathogenesis by reducing the levels of immunosuppressive adenosine (Becker et al., 2019a). Down regulation of ADA activity is associated with increased anti-inflammatory Th2 response, whereas its up-regulation may promote Th1-dependent pro-inflammatory response. Compared to healthy control subjects, SLE patients present significantly higher levels of IL-6, IL-17, IL-12, and IL-23 (Talaat et al., 2015; Furini et al., 2019), which correlate positively and significantly with SLE disease activity index (SLEDAI) score (Talaat et al., 2015). Treg cells from SLE patients express lower levels of NTPDase1/CD39 than Tregs from control subjects, and nearly absent adenosine-dependent Treg-mediated suppression. Therefore functional Treg defects, rather than reduced Treg number, seem to be relevant for loss of peripheral tolerance in SLE (Loza et al., 2011).

Increasing evidence indicates that adenosine and its receptors are protective in SLE. In MRL/lpr mice, a murine model of lupus nephritis, treatment with A_2A_ adenosine receptor agonists significantly reduces proteinuria, blood urea and creatinine as well as serum level of anti-dsDNA antibodies. Moreover, kidney histology is improved following treatment with A_2A_ adenosine receptor agonists, which decreases infiltration of macrophages and T-cells expressing lower MCP-1, IFN-γ and MHC-II levels, and reduces IC deposition (Zhang et al., 2011). Evidence that adenosine might be beneficial in lupus nephritis is supported by the finding that CD39^*−/−*^ or CD73^*−/−*^ mice are more sensitive to pristane-induced lupus-like nephritis compared to WT mice (Knight et al., 2018). Expansion of activated B and plasma cells is found in CD73^*−/−*^ mice, while expansion of Th17 cells is present in mice deficient of either ecto-enzymes. CD39^*−/−*^ and CD73^*−/−*^ mice also exhibit endothelial dysfunction and exaggerated release of extracellular traps (NETs) from neutrophils, while CD73^*−/−*^ mice have higher levels of circulating cell-free DNA (Knight et al., 2018).

In SLE patients, defective NTPDase1/CD39 expression and impaired Treg functions (Loza et al., 2011) are associated with A_2A_ adenosine receptor upregulation in peripheral lymphocytes. A_2A_ adenosine receptor expression directly correlates with SLEDAI index (Bortoluzzi et al., 2016). A_2A_ adenosine receptor agonists lower blood levels of inflammatory cytokines (IFN-α, TNF-α, IL-2, IL-6, IL-1β) and potentiates release of the anti-inflammatory IL-10 (Bortoluzzi et al., 2016). Thus, the use of A_2A_ adenosine receptor agonists might be of therapeutic relevance in SLE (Bortoluzzi et al., 2016).

### Inflammatory Bowel Diseases

Inflammatory bowel diseases (IBD) are a group of chronic inflammatory intestinal disorders including Crohn’s disease (CD), that affect the whole digestive system, mainly the small intestine, and ulcerative colitis (UC), that mainly affects colon and rectum. In IBD, gut wall is heavily infiltrated by immune cells promoting inflammation and tissue damage. IBD etio-pathogenesis is largely unknown, but dysregulated interaction between digestive mucosa and microbiota, together with individual and genetically-determined susceptibility, are invoked to explain disease onset and perpetuation. An imbalance between cellular and humoral immunity to microbiota, characterized by loss of mucosal T-cell-mediated barrier immunity and uncontrolled antibody response, has been recently described (Noble et al., 2019). IBD predisposes to a wide range of complications such as thrombophilia and chronic debility, as well as bowel, lymphatic, and liver cancers. CD and UC show distinct purine gene dysregulation signatures associated with inflammation-related signalling pathways, a finding potentially relevant for the design of novel specific therapeutic approaches (Rybaczyk et al., 2009).

Key players in IBD are type 1 regulatory T (Tr1) and Th17 lymphocytes. Th17 cell maturation and function in the small intestine is controlled by luminal ATP level, which in turn is set by NTPDase7 expressed on mucosal epithelial cells, as demonstrated by increased number of Th17 cells in the small intestinal lamina propria in *Entpd7* ^*−/−*^ mice (Kusu et al., 2013). In the gut, UCB may act as a potent immune modulator since its binding to AhR upregulates NTPDase1/CD39 expression thus leading to immunosuppression (Jangi et al., 2013). Expansion of NTPDase1/CD39^+^ regulatory-type T helper 17 (SupTh17) cells as well as Tr1 cells, which express high levels of IL-10, might be promoted by AhR activation. Reduced NTPDase1/CD39 expression levels and/or dysfunction of AhR abrogate the protective effects of UCB in experimental colitis in mice and in IBD patients. Promising strategies to overcome Th17 dysfunction in IBD might be represented by use of the natural/endogenous AhR ligands to improve immunosuppressive signalling via increased NTPDase1/CD39 expression. A protective role of NTPDase1/CD39 in CD is suggested by evidence originating from patients and from experimental models of colitis in mice. In humans, NTPDase1/CD39 expression by peripheral blood Treg cells is lower in patients with active IBD than in healthy subjects (Gibson et al., 2015). NTPDase1/CD39 expression by Treg cells increased significantly after pharmacological treatment in patients responsive to therapy with clinical and endoscopic remission of the disease (Gibson et al., 2015). In addition, a single nucleotide polymorphism associated with low levels of NTPDase1/CD39 expression is associated with increased susceptibility to CD in a case-control cohort (Friedman et al., 2009). In a murine (CD45RB) T-cell transfer model of colitis, Treg cells with genetic deletion of NTPDase1/CD39 showed reduced ability to suppress intestinal inflammation compared to WT Treg cells (Gibson et al., 2015). Finally, CD39^*−/−*^ mice, compared to WT, are highly susceptible to dextran sodium sulphate (DSS) injury, an experimental model of colitis, while heterozygous mice showed an intermediate phenotype (Friedman et al., 2009). The role of NTPDase1/CD39 in IBD is solid and further confirmed by the finding that NTPDase1/CD39 polymorphisms are associated with IBD in humans and that NTPDase1/CD39 deficiency exacerbates murine colitis (Friedman et al., 2009). On the other hand, despite evidence showing that MTX and sulfasalazine, two drugs currently used to treat IBD, act by stimulating NT5E/CD73-dependent adenosine production (Ochoa-Cortes et al., 2014), the role of NT5E/CD73 is still unclear.

An increased number of NT5E/CD73^+^ CD4^+^ T cells is found in the peripheral blood and in the intestinal *lamina propria* of patients with active IBD, especially during active inflammation. These peripheral NT5E/CD73^+^ CD4^+^ T cells predominantly express CD45RO, are enriched with IL-17A^+^ cells and express high levels of IL-17A and TNF (Doherty et al., 2012). NT5E/CD73 expression is increased by TNF-α and decreased by anti-TNF-α monoclonal antibody. NT5E/CD73^+^ CD4^+^ T cells might represent a novel memory-effector cell population, particularly enriched with Th-17^+^ cells, which could be used to monitor IBD activity during treatment (Doherty et al., 2012).

In chronic DSS-induced colitis, adoptive transfer of GM-CSF activated monocytes (GMaM) leads to substantial clinical improvement, as demonstrated by reduction of weight loss, inflammatory infiltration, ulceration, and colon shrinkage. Compared with control monocytes, GMaM express higher levels of NTPDase1/CD39 and NT5E/CD73, migrate faster and persist longer in the inflamed intestine, thus inducing a more efficient Treg cells generation (Weinhage et al., 2015). While NTPDase1/CD39 expression on Treg cells behaves as a heritable trait shaping adaptive immune response (Roederer et al., 2015), altered Treg NT5E/CD73 expression seems to be more extensively affected by environmental factors such as pathogens, diet or microbiome components (Mangino et al., 2017). Considering the immunosuppressive effect of adenosine, the use of P1R agonists might be a reasonable approach to IBD therapy. Agonists of the A_2A_ adenosine receptor suppress the production of pro-inflammatory cytokines such as IL-2, IFN-γ, and TNF-α, but not the anti-inflammatory cytokines IL-10 and TGF-β, and attenuate experimental colitis in mice (Naganuma et al., 2006). In addition, A_2A_ adenosine receptor activation by endogenously generated adenosine from stimulated myenteric neurons results in a tonic facilitator effect in the gastrointestinal tract (Vieira et al., 2009) likely modulating IBD progression.

In a different animal model of IBD, i.e. post-inflammatory ileitis following 2,4,6-trinitrobenzenesulfonic acid (TNBS)-treatment, lack of adenosine increase following ATP release into the inflamed tissue is hypothesised to be at least partially due to feed-forward inhibition of muscle-bound NT5E/CD73 by excess ATP/ADP (Vieira et al., 2014; Vieira et al., 2017).

In the intestinal mucosa of patients with active UC, expression of genes involved in purine metabolism is modified and associated with up-modulation of group 3 innate lymphoid cell (ILC3)-IL-22 gene pathway. In this context, the NTPDase-mediated ATP/adenosine balance is suggested to regulate ILC3 cell function as a protection against intestinal injury (Crittenden et al., 2018).

### Type 2 Diabetes

Chronic inflammation is an important determinant of insulin resistance, one of the fundamental features of type 2 diabetes (T2D). T2D is a complex disease typical of aged, obese, people, affected by metabolic syndrome. T2D that involves β-cells in pancreatic islets, adipocytes, hepatocytes, muscle cells and many other tissues, arises subtly becoming manifest when insulin resistance is accompanied by impaired insulin secretion. Stimulated and inflamed adipocytes are shown to release ATP (Tozzi et al., 2020), and the increased extracellular ATP concentration is suggested to impair functions of β-cells in pancreatic islets. In β-cells, signalling activated by P2YRs and P2XRs engagement has been implicated in insulin secretion. However, it is not clear whether high ATP levels impair β-cell function directly, e.g., via interaction with P2YRs or P2XRs, or through excessive systemic cytokine release. P2XRs, notably P2X7R, are suggested to play a relevant role in T2D pathogenesis due to their ability to trigger inflammasomes activation and release of inflammatory cytokines (Novak and Solini, 2018; Solini and Novak, 2019). In addition, impaired glucose tolerance and decreased insulin sensitivity is associated with higher plasma insulin levels and altered hepatic glucose metabolism in CD39^*−/−*^ mice (Enjyoji et al., 2008; Chia et al., 2012). The same effects are obtained by administration of either exogenous ATP or ectonucleotidase inhibitors to WT mice, and by *in vitro* exposure of hepatocytes to ATP (Enjyoji et al., 2008). These findings further support the pro-inflammatory effect of the increased extracellular ATP levels that accumulate in absence of NTPDase1/CD39. Increased expression of NTPDase1/CD39 and decreased expression of NT5E/CD73 is found in different lymphocyte subpopulations from T2D obese patients compared to healthy subjects. In addition, NT5E/CD73 blood levels negatively correlate with age, body mass index (BMI), fasting plasma glucose (FPG), glycated haemoglobin (HbAc1), triglycerides and cholesterol (Guzman-Flores et al., 2015). In T2D, a role has been proposed also for Th17 cells that are usually suppressed by NTPDase1/CD39^+^ Treg cells.

In T2D obese patients significantly lower blood level of NTPDase1/CD39+ Treg cells and a negative correlation between NTPDase1/CD39^+^ Treg cells, weight and BMI is found (Cortez-Espinosa et al., 2015). On the other hand, low levels of CD4^+^ IL-17^+^ cells in overweight and obese T2D patients positively correlates with glucose and HbA1c (Cortez-Espinosa et al., 2015), whereas a subpopulation of SupTh17 NTPDase1/CD39+ cells negatively correlates with glycemia and HbA1c. On the whole, these findings indicate a relationship between NTPDase1/CD39 expression on both Treg and CD4^+^ IL-17^+^ cells and hyper-glycemia, overweight and obesity (Cortez-Espinosa et al., 2015). Finally, it is an established fact that adenosine receptor blockade reverses insulin resistance in skeletal muscle from diabetic rats (Challis et al., 1984), and pharmacological manipulation of the adenosinergic system is proposed as an approach to manage T2D and associated complications (Antonioli et al., 2015; Deb et al., 2019).

## Tumor Inflammatory Environment

Ectonucleotidases regulate inflammation in different pathophysiological contexts, but on the other hand, the inflammatory environment can influence ectonucleotidases expression and function. It is well established that inflammation is a feature of the TME and that purinergic signalling and ectonucleotidase products play a role in cancer growth and tumor-host interactions (Adinolfi et al., 2015; Di Virgilio and Adinolfi, 2016; Allard et al., 2019). In the TME, extracellular ATP levels are increased (Pellegatti et al., 2008; Di Virgilio et al., 2018b) likely promoting inflammation and anti-tumor immune response, whereas adenosine is chiefly responsible of dysregulation of immune cell infiltrate resulting in tumor progression and metastatic spreading (Young et al., 2014; Mittal et al., 2016; Sitkovsky, 2020).

Among ATP receptors, the P2X7R is the subtype most convincingly associated to tumor growth (Adinolfi et al., 2012; Giuliani et al., 2014; Amoroso et al., 2016), and at the same time involved in the modulation of NTPDase1/CD39 and NT5E/CD73 expression in the TME (De Marchi et al., 2019). The immune infiltrate in B16F10 mouse melanoma tumors growing in the syngeneic P2×7^−/−^ host shows clear-cut immunosuppressive features, which are absent in the immune infiltrate from same tumors growing in WT mice. CD8^+^ cells are decreased while Treg cells are increased and overexpress the fitness markers OX40 and PD-1 (De Marchi et al., 2019). Tregs overexpress NT5E/CD73 while Teff cells overexpress both NT5E/CD73 and NTPDase1/CD39. Increased NT5E/CD73 in P2×7^−/−^ mice is paralleled by a decrease in the TME ATP concentration. The immunosuppressive signature is confirmed by the faster growth of tumors implanted in the P2×7^−/−^ host compared to WT (De Marchi et al., 2019). An increase in NT5E/CD73 expression is also found in Tregs from the spleen of P2×7^−/−^ tumor-bearing mice. The immunosuppressive signature in P2×7^−/−^ is confirmed by the switch of systemic cytokines to an anti-inflammatory profile characterized by increased TGF-β and decreased IL-1β, TNF-α, and IFN-γ plasma levels (De Marchi et al., 2019)*.* Systemic administration of a P2X7R antagonist to tumor-bearing WT mice reduces tumor growth and upsets the immune infiltrate causing on one hand an increase in CD4^+^ and Teff cells, and on the other a down-modulation of both NTPDase1/CD39 and NT5E/CD73 expressed by CD8^+^ Treg cells (De Marchi et al., 2019).

Another relevant player in the TME are tumor-associated macrophages (TAMs). TAM NTPDase1/CD39 expression is increased by AhR recruitment via glioblastoma cells products, such as kynurenine. Adenosine produced in cooperation with NT5E/CD73 promotes CD8^+^ T cell dysfunction (Takenaka et al., 2019). Human grade 4 gliomas indeed show highest AhR and NTPDase1/CD39 expression and elevated AhR expression level is associated with poor prognosis (Takenaka et al., 2019).

Anti- NTPDase1/CD39 antibodies inhibiting conversion of extracellular ATP to AMP show potent anti-tumor activity since they do not only reduce adenosine concentration but also trigger the ATP-P2X7R-NLRP3 inflammasome-IL-18 axis. Active IL-18 release facilitates expansion of intra-tumor effector T cells whereas intra-tumor macrophages are reduced (Li et al., 2019). Anti-NTPDase1/CD39 antibodies facilitate intra-tumor T cell infiltration overcoming resistance to PD-1 blockade, therefore showing potentially useful activity in the adoptive T-cell transfer therapy (Li et al., 2019).

In models of tumor metastases, NTPDase1/CD39 is expressed on tumor-infiltrating Treg cells, myeloid cells and some NK cell subtypes. NK cell number and function is increased in NTPDase1/CD39-deficient mice, as well as in WT mice treated with the NTPDase inhibitor sodium polyoxotungstate (POM-1). POM-1 is an effective inhibitor of experimental and spontaneous metastases in several different tumor models, and its action is fully abrogated in mice with NK cells depletion, IFN-γ neutralization or deficient NTPDase1/CD39 expression in bone marrow-derived cells (Zhang et al., 2019). The development of NTPDase1/CD39-based therapies appears particularly relevant in the perspective to inhibiting the NTPDase1/CD39 pathway and the related NK cell-mediated anti-tumor immunity suppression (Zhang et al., 2019). Since high levels of NT5E/CD73 expression on tumor cells are significantly associated with reduced disease free survival (DFS) and overall survival (OS), and negatively correlate with tumor infiltration by immune cells, NT5E/CD73 targeting could be a promising strategy to reprogram the TME (Buisseret et al., 2018).

A combination of drugs targeting NT5E/CD73 and the A_2A_ adenosine receptor has been shown to potentiate anti-tumor immune responses decreasing tumor growth and metastatic spreading (Young et al., 2016). To promote an *in vivo* optimal therapeutic response, effector lymphocytes, IFN-γ and anti-NT5E/CD73 antibodies engaging activating Fc receptors are required. Fc receptor binding indeed augment the production of proinflammatory cytokines that potentiates the immune response (Young et al., 2016).

On the basis of these observations, extracellular adenosine can be considered a *bona fide* "immune checkpoint mediator" (Allard et al., 2017; Boison and Yegutkin, 2019). Targeting NTPDase1/CD39, NT5E/CD73, adenosine or adenosine receptors is increasingly recognized as a promising intervention in anti-cancer therapy (Young et al., 2016; Allard et al., 2017).

## Conclusion

As any homeostatic process, inflammation must be tightly controlled to fulfil its scope, i.e. removal of endogenous and exogenous injurious agents to restore tissue integrity. Regulation of acute and chronic inflammatory responses is thus critical to preserve good health. Ectonucleotidases play a major role by setting the balance between pro-inflammatory nucleotides and anti-inflammatory adenosine. The main mechanism responsible for the accumulation of ATP into the extracellular space is transport across the plasma membrane, therefore the different transport pathways involved also play a crucial role in regulating ectonucleotidases activity. Virtually, responses of all immune cells, e.g., neutrophils, monocytes/macrophages, various T lymphocyte subsets and B lymphocytes, are affected to a larger or smaller extent by NTPDase1/CD39 and NT5E/CD73. The different acute and chronic inflammatory conditions, tumor-related inflammation included, briefly explored in this review with particular attention to more recent findings, demonstrate the relevant role of the ectonucleotidases in inflammatory homeostasis.
